# Assessing Knowledge and Attitudes towards Cervical Cancer Screening among Rural Women in Eastern China

**DOI:** 10.3390/ijerph14090967

**Published:** 2017-08-27

**Authors:** Tongtong Liu, Shunping Li, Julie Ratcliffe, Gang Chen

**Affiliations:** 1 School of Health Care Management, Shandong University, Jinan 250012, China; liutongtong23@163.com; 2 Key Laboratory of Health Economics and Policy Research, NHFPC (Shandong University), Jinan 250012, China; 3 Institute for Choice, Business School, University of South Australia, Adelaide 5000, Australia; Julie.Ratcliffe@unisa.edu.au; 4 Flinders Centre for Innovation in Cancer, College of Medicine and Public Health, Flinders University, Adelaide 5042, Australia; gang.chen@flinders.edu.au

**Keywords:** cervical cancer, knowledge, attitude, China

## Abstract

There is a heavy burden of cervical cancer in China. Although the Chinese government provides free cervical cancer screening for rural women aged 35 to 59 years, the screening rate remains low even in the more developed regions of eastern China. This study aimed to assess knowledge and attitudes about cervical cancer and its screening among rural women aged 30 to 65 years in eastern China. A cross-sectional study was conducted in four counties of Jining Prefecture in Shandong Province during August 2015. In total, 420 rural women were randomly recruited. Each woman participated in a face-to-face interview in which a questionnaire was administered by a trained interviewer. A total of 405 rural women (mean age 49 years old) were included in the final study. Among them, 210 (51.9%) participants had high knowledge levels. An overwhelming majority, 389 (96.0%) expressed positive attitudes, whilst only 258 (63.7%) had undergone screening for cervical cancer. Related knowledge was higher amongst the screened group relative to the unscreened group. Age, education and income were significantly associated with a higher knowledge level. Education was the only significant factor associated with a positive attitude. In addition, women who were older, or who had received a formal education were more likely to participate in cervical cancer screening. The knowledge of cervical cancer among rural women in eastern China was found to be poor, and the screening uptake was not high albeit a free cervical cancer screening program was provided. Government led initiatives to improve public awareness, knowledge, and participation in cervical cancer screening programs would likely be highly beneficial in reducing cervical cancer incidence and mortality for rural women.

## 1. Introduction

Cervical cancer ranks as the fourth most common cancer in women worldwide [1]. There were an estimated 528,000 new cases and 266,000 deaths from cervical cancer in 2012, and around 85% of these occurred in less developed regions [1]. Cervical cancer is mainly caused by persistent infection with certain types of human papillomavirus (HPV). HPV types 16 and 18 are responsible for approximately 70% of cervical cancer cases in all countries around the world [2]. Other risk factors include onset of intercourse before 20 years old, having multiple sexual partners, and smoking [3,4]. Cervical cancer is responsible for a heavy economic and social burden, and its incidence and mortality have increased from 2003 to 2010 in China [5]. It is estimated that 98,900 new cancer cases and 30,500 cancer deaths occurred in China in 2015 alone [6].

Almost all women may be infected with HPV in their lifetime, but in most cases, HPV infections will be cleared by the body’s immune response without treatment [7]. HPV infections may lead to cervical cancer and may be completely asymptomatic in the early stage; women can live in a pre-cancerous state for 10–15 years without any symptoms [8]. Fortunately, cervical cancer is a preventable and treatable disease; screening of precancerous lesions can reduce its incidence and mortality. The major screening techniques include Papanicolaou smear, visual inspection using acetic acid, and HPV tests; the latter two of which have been found to be cost-effective for low-resource countries [9]. The World Health Organization (WHO) recommends that all women between the ages of 30 and 49 years should be screened for cervical cancer at least once [8]. In America, where cervical cancer screening programs have become well established, annual incidence has declined by 75% or more over the past half century [10].

Cervical cancer remains a major health problem in Chinese women especially for women living in rural China. In order to reduce this burden, China’s government launched the National Cervical Cancer Screening Program in Rural Areas (NCCSPRA) in 221 pilot counties commencing in 2009. A total of 11.69 million rural women between 35 and 59 years old accessed this screening program from 2009 to 2011 [11]. A lack of knowledge about cervical cancer screening among rural women in China has been highlighted as one of the most important factors influencing participation rate of screening [11]. Other barriers to accessing cervical cancer screening included anxiety about the possibility of being diagnosed with cervical cancer and the lack of symptoms [12]. The Jining government has provided free breast cancer and cervical cancer screening for rural women aged 35 to 59 years since 2011 and the upper age limit for the target population was extended to 64 years in 2014.

Substantive evidence relating to knowledge, attitudes and participation rates for rural Chinese women in cervical cancer screening is currently lacking. Therefore, the main aim of this study was to assess the knowledge and attitudes towards cervical cancer screening among rural women in Jining Prefecture and to provide valuable information to facilitate policy-making in relation to the provision and uptake of cervical cancer screening in China.

## 2. Materials and Methods

### 2.1. Study Design

A stratified random sampling framework was employed for the study design. Among 11 counties in Jining Prefecture, four counties (Rencheng County, Qufu County, Sishui County and Yutai County) were randomly selected. Two townships were randomly selected within each county and then two villages were randomly selected from within each sampled township. In the final step, women were randomly selected from potential candidate participants identified by each village committee. No less than 100 participants per county were interviewed to increase the representative of each county. Women aged 30 to 34 years are the potential screening populations in the future, and women aged 65 years were still the target populations when the second wave of cervical cancer screening was conducted in 2014. Therefore, women who were outside the target age range (30 to 65 years), women who had previously had a hysterectomy, or who indicated no prior sexual experience were excluded from this study.

This study was approved by the Medical Ethics Committee of Medical School, Shandong University (LL-201401048), and conforms to the ethics guidelines of the Declaration of Helsinki. Informed consent was obtained from all participants prior to questionnaire administration.

### 2.2. Data Collection

A pilot investigation, including 35 rural women from four counties (Rencheng County, Qufu County, Sishui County and Yutai County) located in Jining Prefecture, was carried out to examine intelligibility, acceptability, and validity of the questionnaire in June 2015 prior to the main study in August 2015. Five researchers from Shandong University were trained to administer the questionnaire. As the majority of participants had relatively low educational levels, the questionnaire was administered as a face-to-face interview to facilitate participant understanding and completeness and thereby ensure the quality of the investigation.

The questionnaire consisted of three main sections. The first section contained a series of socio-demographic questions. The second section include 17 items, including 7 items about common cervical cancer knowledge, 6 items about risk factors and symptoms of cervical cancer, and 4 items about cervical cancer screening knowledge. Following previous literature, each correct response was scored as 1, with incorrect or “do not know” responses scored as 0, and a cutoff point of 50% correction rate was used to categorize respondents into 2 groups: the total score ranging from 9 to 17 was defined as a high level of knowledge, and the total score ranging from 0 to 8 was defined as a low level of knowledge [13,14]. The third section of the questionnaire assessed rural women’s attitudes towards cervical cancer and its screening. Those women whose answers were “it’s necessary to disseminate related knowledge of cervical cancer” and “I am supportive of a cancer screening program” were regarded as having a positive attitude.

The process of administering the questionnaire took 20–30 min on average and all completed questionnaires were returned directly to the investigators.

### 2.3. Data Analysis

Responses were coded and double-entered into an electronic database using EpiData version 3.1 (EpiData Association, Odense, Denmark). All statistical analyses were performed using the IBM SPSS Statistics version 21 (IBM, Chicago, IL, USA). Categorical variables were summarized as frequencies and percentages, and continuous variables as means and standard deviation. The chi-square test was used to assess the differences of socio-demographic characteristics, the related knowledge on cervical cancer and its screening between screened and unscreened rural women. Binary logistic regression was used to separately assess the associations of the adequacy of knowledge and attitudes towards cervical cancer and cervical cancer screening with sociodemographic characteristics. All variables associated with *p*-values < 0.1 according to univariate analysis were included in the logistic regression models. The results of binary logistic regression analyses were reported as odds ratios (OR) and their 95% confidence intervals (95% CI). *p*-values < 0.05 were regarded as statistically significant.

## 3. Results

A total of 420 rural women provided informed consent to participate. Among them, 15 (3.6%) women were excluded based on exclusion criteria. The final sample consisted of 405 rural women in total from four counties.

The key socio-demographic characteristics of participants are presented in Table 1. The mean age of participants was 49.2 ± 8.3 years (age range: 30 to 65 years), and almost 50% were aged 45 to 54 years. Around 3.7% (*n* = 15) women were divorced or widowed. On average, 27.4% (*n* = 111) of participants never went to school and this proportion was much higher in Sishui County (50.5%). Approximately half (51.4%) of participants indicated an annual family income less than RMB 20,000 (US$3212) and on average it was found that participants from Sishui County were the poorest.

The key socio-demographic characteristics among rural women of screened and unscreened groups are presented in Table 2. As can be seen, education level was the only statistically significant characteristic that differs between the two groups, with the women in the screened group having higher education than women in the unscreened group.

A summary of knowledge levels in relation to key aspects of cervical cancer and its screening between screened and unscreened groups is presented in Table 3. The mean knowledge scores of the screened group (258 women) and the unscreened group (147 women) were 9.29 ± 2.64 (range from 3 to 16 score) and 5.03 ± 4.71 (range from 0 to 15 score), respectively. There were statistically significant differences between the screened and unscreened groups in all items except for the item “women should be screened at least every three years”, with the screened group having better knowledge. Notable variations were evident across the study sample in relation to specific knowledge items; with knowledge levels in relation to how many years one could be expected to live following active treatments, risk factors, symptoms of cervical cancer, and available screening methods being the poorest. For instance, only 23.3% (*n* = 60) of the screened women and 8.8% (*n* = 13) of unscreened women indicated knowledge that cervical smear cytological examination is a major method of undertaking cervical cancer screening.

The main findings in relation to the adequacy of knowledge and attitudes towards cervical cancer and cervical cancer screening are summarized in Table 4. It can be seen that approximately half (51.9%) of the study sample were classified with the higher knowledge level. Although the vast majority (96.0%) of women expressed a positive attitude towards cervical cancer screening, only 258 (67.3%) of participants indicated that they had previously undergone cervical cancer screening.

Table 5 presents the results of three binary logistic regression models to identify the main predictors of adequacy of knowledge and attitudes towards cervical cancer and its screening. Age, educational level and family income were significantly associated with a higher knowledge level. Knowledge levels in older women (aged 55–65 years) (OR = 0.48; 95% CI: 0.26–0.90) were lower in general than women aged 30–44 years. Women reporting themselves with higher educational levels, having completed middle school or a higher level of education (OR = 3.47; 95% CI: 2.00–6.02) were more likely to have sufficient knowledge. Women with incomes of over RMB 10,000 (US$1606) a year were found to have adequate knowledge. Educational level was the only factor found to be significantly associated with positive attitudes. The attitudes of women who had completed middle school or a higher level of education (OR = 11.25; 95% CI: 1.33–95.20) were more positive compared with women who indicated that they had never attended school. Women who had attended primary school (OR = 3.31; 95% CI: 1.85–5.93), or attended middle school (OR = 4.82; 2.72–8.56) were more likely to attend screening than those who never went to school. Women aged 45–65 years were more likely to have undergone cervical cancer screening than women aged 30–44 years.

## 4. Discussion

Cervical cancer is preventable and curable at early stages [8]. A total of 15.3% of the participants in this study indicated that they had never heard of cervical cancer previously, a similar rate to a study conducted in Italy in 2008 (17.0%) [15]; however, this rate is lower than that found in other previous Chinese studies [16,17]. The difference in findings may potentially be attributable to the provision of free cervical cancer screening for rural women and related public education programs which have been offered by the Jining government since 2011. Whilst the vast majority of rural Chinese women participating in this study had heard of cervical cancer screening previously, the findings from this study indicate that awareness rates in relation to cervical cancer and its screening amongst rural women in Jining province were quite limited. Only 51.9% of rural women exhibited higher knowledge levels, a similar rate to a previous study which was conducted in Kuwaiti [18], but higher than the findings from a similar study conducted in Congo (43%) [19]. This finding is important because previous studies have found a strong link between knowledge about cervical cancer and its screening and participation rates [12,15].

The overwhelming majority (96.0%) of participants expressed a positive attitude towards screening, while only 63.7% women had undergone cervical cancer screening. These results are similar to those reported in other studies [13,20]. It was apparent in this study that the levels of knowledge in the screened group were higher than that of the unscreened group. Lower levels of knowledge may be related to insufficient health education, limited access to relevant information to increase knowledge, lack of communication between rural women and health service providers, and some cultural barriers [21]. Public education programs which aim to increase knowledge and awareness of cervical cancer and the benefits of screening have been found to be an effective method to increase screening uptake, as well as increase the likelihood of maintaining regular screening behaviors [22]. The women in rural China have relatively lower educational levels [23] and a few women told us during the face-to-face interview survey that they did not understand and/or were not willing to read the cervical cancer information booklet provided by the government. Instead, they preferred to attend free face-to-face seminars on cervical cancer prevention and control provided by health care workers, or watch relevant health promotion videos. Indeed, a multimedia health education program has been found to effectively improve the knowledge and awareness of cervical cancer and its screening, and also increased the uptake of screening services among adult rural women in Nigeria [24].

The role of health service providers has been found to be crucial in the health promotion of cervical cancer knowledge and screening [25], hence the knowledge that health service providers have about cervical cancer plays an important role in delivering accurate and adequate information to women. A survey conducted in China in 2013 found disturbing results that in fact the knowledge level of health care workers who provide cervical cancer screening was generally low with an overall combined knowledge rate of only 46.9% [25]. Health promotion and education on cervical cancer knowledge should therefore not only target women but also health care workers. Continuing education for health care workers should be provided so that the frontline health workers are equipped with up to date knowledge and skills. In addition, mechanisms should be explored and established such that health workers (including nurses and midwives) have the capacity to provide sustainability in their health education activities, since a single brief health talk may not increase the screening uptake [26,27]. Special budget allowance could be decentralized towards primary health care facilities to accommodate health education activities. The Central government has increased the annual basic public health services (including health education) subsidy to RMB 45 (US$7) per person in 2017 [28], and future study should evaluate the effectiveness of increasing the budget on health knowledge and attitudes.

Consistent with previous studies, this study found that age, level of education and income were significantly associated with a higher knowledge level [11,13]. Younger women with higher education and income levels were more likely to have adequate knowledge. It is possible that younger women pay more attention to their health and have more opportunities to obtain relevant information and thereby increase their knowledge. In accordance with a previous international study [18], level of education was the only significant factor associated with positive attitudes, and age and level of education were significantly associated with positive participation rates. Our study showed that older women were significantly more likely to attend cervical cancer screening than younger women. This observation cannot be explained by the knowledge level as younger women have a higher level of knowledge. A higher opportunity cost to attend cervical cancer screening for younger women could be the potential reason. Younger women are more likely to be working full time and/or taking care of younger children at home. As such they were less likely to take leave to attend screening as compared to older women who are usually more flexible with their time. A follow up in-depth qualitative study would be helpful to further elucidate the reasons behind this observation.

HPV vaccines are highly effective in preventing persistent HPV infection and the subsequent precancerous lesions due to infection with two types of HPV (known as types 16 and 18) [29]. As an important primary prevention intervention against cervical cancer, HPV vaccination of girls should occur prior to onset of sexual activity [29]. So far, HPV vaccination is not yet routinely available for women in mainland China. Cervical cancer screening is an effective method for reducing incidence and mortality of cervical cancer. In this study, over one third (36.3%) of rural Chinese women indicated that they had never undergone cervical cancer screening. Previous studies have indicated that the main barriers to participation in cervical cancer screening include a lack of knowledge and awareness of cervical cancer screening and its benefits, shortage of staff, equipment and supplies, the fear of pain and being diagnosed with cervical cancer, embarrassment, the lack of husband’s support for screening, and cultural factors [21,30,31,32]. Chinese governments currently provide free cervical cancer screening for target populations in pilot counties, but in the longer-term women may be asked to contribute towards the cost of providing these services which will become an additional barrier for many rural Chinese women. Incorporating cervical cancer screening programs into medical insurance programs may be an effective method to increase provision and access to cervical cancer screening programs in the future. 

### Limitations

This study has some limitations. Firstly, this study focused on the knowledge and attitudes of rural women from Jining Prefecture in Shandong Province. Consequently, the findings may not be generalizable to other regions of Eastern China. Secondly, the potential role of men in improving cervical cancer screening rates was not investigated in this study. Thirdly, the questionnaire utilized in this study was designed for the collection and analysis of quantitative data only. A qualitative in-depth follow on study conducted in this population group would be helpful in providing richer data to supplement the summary results and quantitative data presented here. 

## 5. Conclusions

This study found that the overwhelming majority of Chinese rural women in eastern China expressed positive attitudes towards cervical cancer screening. However, many women had low levels of knowledge and over one third had never participated in a cervical cancer screening program. Public education programs are likely to provide a very effective method for women and health workers to increase their knowledge, awareness, and utilization of cervical cancer screening to reduce cervical cancer incidence and mortality.

## Figures and Tables

**Table 1:** Socio-demographic characteristics of the participants in the four counties, *n* (%).

Demographic Characteristics	County				
	Qufu	Yutai	Sishui	Rencheng	Total
**Age (years)**					
30–44	27 (26.0%)	24 (23.8%)	31 (31.3%)	28 (27.7%)	110 (27.2%)
45–54	41 (39.4%)	45 (44.5%)	46 (46.5%)	53 (52.5%)	185 (45.6%)
55–65	36 (34.6%)	32 (31.7%)	22 (22.2%)	20 (19.8%)	110 (27.2%)
**Marital status**					
Married	100 (96.2%)	99 (98.0%)	96 (97.0%)	95 (94.1%)	390 (96.3%)
Divorced or widowed	4 (3.8%)	2 (2.0%)	3 (3.0%)	6 (5.9%)	15 (3.7%)
**Educational level**					
No school	21 (20.2%)	26 (25.7%)	50 (50.5%)	14 (13.8%)	111 (27.4%)
Primary school	25 (24.0%)	35 (34.7%)	31 (31.3%)	33 (32.7%)	124 (30.6%)
Middle school	47 (45.2%)	31 (30.7%)	13 (13.1%)	43 (42.6%)	134 (33.1%)
High school or above	11 (10.6%)	9 (8.9%)	5 (5.1%)	11 (10.9%)	36 (8.9%)
Family income * (RMB (US$) per year)					
<10,000 (1606)	14 (13.5%)	20 (19.8%)	60 (60.6%)	10 (9.9%)	104 (25.7%)
10,000–20,000 (1606–3212)	21 (20.2%)	28 (27.7%)	19 (19.2%)	36 (35.6%)	104 (25.7%)
20,000–30,000 (3212–4818)	28 (26.9%)	28 (27.7%)	10 (10.1%)	25 (24.8%)	91 (22.4%)
>30,000 (4818)	41 (39.4%)	25 (24.8%)	10 (10.1%)	30 (29.7%)	106 (26.2%)
**Total**	104 (25.7%)	101 (24.9%)	99 (24.5%)	101 (24.9%)	405 (100%)

According to the Organisation for Economic Co-operation and Development (OECD) data (https://data.oecd.org/conversion/exchange-rates.htm), the average annual exchange rate between US$ and RMB in 2015 was: US$1 = RMB 6.227. * Per capita disposable income of rural residents in China was RMB 11,422 (US$1834) (Year 2015). Per capita disposable income of rural residents in Shandong Province was RMB 12,930 (US$2077) (Year 2015).

**Table 2:** Socio-demographic characteristics between screened and unscreened groups, *n* (%).

Demographic Characteristics	Screened Group	Unscreened Group	χ2	*p*-Value
**Age (years)**			4.51	0.11
30–44	64 (24.8%)	46 (31.3%)		
45–54	128 (49.6%)	57 (38.8%)		
55–65	66 (25.6%)	44 (29.9%)		
**Marital status**			0.63	0.43
Married	247 (95.7%)	143 (97.3%)		
Divorced or widowed	11 (4.3%)	4 (2.7%)		
**Educational level**			32.46	0.00
No school	47 (18.2%)	64 (43.5%)		
Primary school	83 (32.2%)	41 (27.9%)		
Middle school	100 (38.8%)	34 (23.1%)		
High school or above	28 (10.8%)	8 (5.5%)		
**Family income (RMB (US$) per year)**			7.13	0.07
<10,000 (1606)	55 (21.3%)	49 (33.3%)		
10,000–20,000 (1606–3212)	71 (27.5%)	33 (22.5%)		
20,000–30,000 (3212–4818)	61 (23.7%)	30 (20.4%)		
>30,000 (4818)	71 (27.5%)	35 (23.8%)		

**Table 3:** Related knowledge of cervical cancer and its screening among rural women of screened and unscreened groups, *n* (%).

	Screened Group	Unscreened Group	χ2	*p*-Value
Have you heard about cervical cancer?	258 (100%)	85 (57.8%)	128.49	0.00
Cervical cancer is not a genetic disease.	162 (62.8%)	39 (26.5%)	49.25	0.00
Cervical cancer has a long precancerous lesions period.	169 (65.5%)	60 (40.8%)	23.23	0.00
Cervical cancer can be detected in its earliest stages.	206 (79.8%)	65 (44.2%)	53.69	0.00
Cervical cancer is curable if detected early.	155 (60.1%)	41 (27.9%)	38.85	0.00
Patients can expect to live 10 more years after active treatments.	87 (33.7%)	18 (12.2%)	22.49	0.00
Postmenopausal women still have the risk of getting cervical cancer.	166 (64.3%)	53 (36.1%)	30.17	0.00
HPV infection is a necessary factor inducing cervical cancer.	99 (38.4%)	34 (23.1%)	9.87	0.00
HPV-positive women may not have cervical cancer.	80 (31.0%)	22 (15.0%)	12.79	0.00
Maintaining sexual hygiene can prevent cervical cancer.	227 (88.0%)	77 (52.4%)	63.41	0.00
Cervical cancer has no symptoms in the precancerous lesions period.	150 (58.1%)	55 (37.4%)	16.09	0.00
Postcoital bleeding is one of the symptoms of cervical cancer.	75 (29.1%)	19 (12.9%)	13.70	0.00
Early sexual activity is one of the risk factors of cervical cancer.	70 (27.1%)	23 (15.6%)	6.98	0.01
Cervical precancerous lesions may be detected by screening.	231 (89.5%)	74 (50.3%)	77.37	0.00
Women should be screened for cervical cancer at least every three years.	7 (2.7%)	1 (0.7%)	1.09	0.30
Cervical smear cytological examination is a major method for cervical cancer screening.	60 (23.3%)	13 (8.8%)	13.16	0.00
The main aim of cervical cancer screening is to discover precancerous lesion early.	195 (75.6%)	61 (41.5%)	46.78	0.00

HPV: Human papillomavirus.

**Table 4:** Adequacy of knowledge, attitude towards cervical cancer and its screening.

	*n*	%
**Knowledge**		
Score > 8	210	51.9%
Score ≤ 8	195	48.1%
**Attitude**		
Positive	389	96.0%
Negative	16	4.0%
**Practice**		
Ever screened	258	63.7%
Never screened	147	36.3%

**Table 5:** Associations of the adequacy of knowledge, attitude towards cervical cancer and its screening with socio-demographic characteristics.

Demographic Characteristics	High Knowledge Level				Positive Attitude				Ever Screened			
	Crude OR (95% CI)	*p*-Value	Adjusted OR (95% CI)	*p*-Value	Crude OR (95% CI)	*p*-Value	Adjusted OR (95% CI)	*p*-Value	Crude OR (95% CI)	*p*-Value	Adjusted OR (95% CI)	*p*-Value
**Age (years)**												
30–44	1	-	1	-	1	-	1	-	1	-	1	-
45–54	0.58 (0.36–0.95)	0.03	0.70 (0.42–1.20)	0.20	0.56 (0.06–5.42)	0.61	0.89 (0.09–9.28)	0.92	1.61 (0.99–2.64)	0.06	2.12 (1.24–3.60)	0.01
55–65	0.31 (0.18–0.54)	0.00	0.48 (0.26–0.90)	0.02	0.08 (0.01–0.59)	0.01	0.25 (0.03–2.31)	0.22	1.08 (0.63–1.85)	0.78	2.22 (1.17–4.20)	0.02
**Marital status**												
Married	1	-			1	-	1	-	1	-		
Divorced or widowed	0.81 (0.29–2.27)	0.68			0.24 (0.05–1.18)	0.08	0.63 (0.11–3.72)	0.61	1.59 (0.50–5.09)	0.43		
**Educational level**												
No school	1	-	1	-	1	-	1	-	1	-	1	-
Primary school	1.79 (1.05–3.05)	0.03	1.31 (0.74–2.33)	0.36	8.09 (1.78–36.71)	0.01	4.01 (0.77–20.94)	0.10	2.76 (1.62–4.69)	0.00	3.31 (1.85–5.93)	0.00
Middle school or above	5.07 (3.02–8.50)	0.00	3.47 (2.00–6.02)	0.00	22.42 (2.89–173.99)	0.00	11.25 (1.33–95.20)	0.03	4.15 (2.48–6.93)	0.00	4.82 (2.72–8.56)	0.00
**Family income (RMB (US$) per year)**												
<10,000 (1606)	1	-	1	-	1	-	1	-	1	-	1	-
10,000–20,000 (1606–$3212)	2.43 (1.38–4.28)	0.00	2.03 (1.12–3.67)	0.02	12.18 (1.54–96.18)	0.02	7.62 (0.93–62.64)	0.06	1.92 (1.09–3.37)	0.02	1.66 (0.91–3.03)	0.10
20,000–30,000 (3212–$4818)	3.60 (1.99–6.51)	0.00	2.38 (1.27–4.47)	0.01	3.47 (0.94–12.85)	0.06	1.43 (0.34–5.92)	0.63	1.81 (1.01–3.24)	0.05	1.40 (0.74–2.63)	0.30
>30,000 (4818)	4.03 (2.27–7.16)	0.00	2.51 (1.35–4.65)	0.00	12.42 (1.57–98.03)	0.02	4.81 (0.57–40.71)	0.15	1.81 (1.03–3.16)	0.04	1.45 (0.78–2.69)	0.24

Crude OR: odds ratio by univariate analysis; Adjusted OR: odds ratio by binary logistic regression models.
